# AMPK/p38 MAPK signaling selectively enhances HIF-induced VEGF-A165 expression under hypoxic and low-glucose conditions in HepG2 cells to promote endothelial cell proliferation and migration

**DOI:** 10.1186/s12885-026-16069-0

**Published:** 2026-05-06

**Authors:** Hirohito Hashinokuchi, Munekazu Yamakuchi, Sadayuki Higashi, Kazunori Takenouchi, Akito Tabaru, Yoko Oyama, Chieko Fujisaki, Kiyonori Tanoue, Masashi Okawa, Fuminori Namino, Teruto Hashiguchi

**Affiliations:** 1 Department of Laboratory and Vascular Medicine, Graduate School of Medical and Dental Sciences, Kagoshima University, 8-35-1, Sakuragaoka, Kagoshima, 890-8544 Japan; 2 Department of Laboratory and Medicine, Kagoshima University Hospital, Kagoshima, Japan; 3 Division of Biobank, Research Support Unit, Center for Advanced Science Research and Promotion, Kagoshima University, Kagoshima, Japan; 4 Department of Digestive Surgery, Graduate School of Medical and Dental Sciences, Kagoshima University, Kagoshima, Japan

**Keywords:** Tumor angiogenesis, Vascular endothelial growth factor, Hypoxia, Glycolysis, Tumor microenvironment, AMP-activated protein kinase, P38 mitogen-activated protein kinase

## Abstract

**Background:**

Angiogenesis is essential for tumor development and growth. Vascular endothelial growth factor-A (VEGF-A), a key regulator of angiogenesis, has several isoforms; however, their expression patterns and mechanisms of action are not fully understood. In this study, we aimed to investigate the expression patterns of VEGF-A isoforms and their effects on endothelial cell responses related to angiogenesis under hypoxic and low-glucose conditions, which are major features of the tumor microenvironment, using a hepatocellular carcinoma cell line (HepG2).

**Methods:**

We cultured human liver cancer-derived cell lines under hypoxic and low-glucose conditions and analyzed the expression patterns of VEGF-A isoforms using an original enzyme-linked immunosorbent assay system that could separately detect human VEGF-A121 and VEGF-A165, which we had previously developed.

**Results:**

The addition of low-glucose conditions to a hypoxic environment increased *VEGF-A* mRNA and protein expression in HepG2 cells, particularly that of VEGF-A165. Since it is known that AMP-activated protein kinase (AMPK)/p38 mitogen-activated protein kinase (p38 MAPK) signaling increases the stability of *VEGF-A* mRNA, we investigated the involvement of this signal and found that activation of AMPK increased VEGF-A165 protein expression, while inhibition of AMPK and p38 MAPK reduced VEGF-A165 protein expression. Furthermore, the effects of VEGF-A isoforms on human umbilical vein endothelial cells (HUVECs) were examined. VEGF-A165 activated phosphorylation signals in HUVECs and upregulated their proliferation and migration abilities compared to VEGF-A121.

**Conclusions:**

These findings suggest that AMPK/p38 MAPK signaling selectively enhances hypoxia-inducible factor-induced VEGF-A165 expression in tumors and promotes endothelial cell proliferation and migration, which may contribute to angiogenesis in vivo.

**Supplementary Information:**

The online version contains supplementary material available at 10.1186/s12885-026-16069-0.

## Background

Tumor cells require substantial energy to sustain rapid growth. Consequently, hypermetabolic tumors with high oxygen consumption, such as hepatocellular carcinoma, are often exposed to hypoxic environments [1]. Under hypoxic conditions, hypoxia-inducible factor-1α (HIF-1α) is stabilized [2], leading to increased glucose uptake through the induction of glucose transporter 1 (GLUT1) [3] and upregulation of glycolysis-related enzymes [4], thereby enhancing glycolytic ATP production as an adaptive response. In contrast, the Warburg effect refers to preferential glycolysis despite the presence of oxygen (aerobic glycolysis) [5]. In addition to hypoxia, tumors frequently experience reduced nutrient availability, including limited glucose, as major features of the tumor microenvironment. These metabolic stresses can induce pro-angiogenic signaling, in which vascular endothelial growth factor (VEGF) plays a central role [6, 7].

Currently, five types of VEGF have been identified: VEGF-A, -B, -C, -D, and placental growth factor [8, 9], among which VEGF-A is primarily responsible for angiogenesis [10]. The *VEGF-A* gene has different isoforms, including *VEGF-A121*, *VEGF-A165*, *VEGF-A189*, and *VEGF-A206*, owing to differences in splicing sites [11]. These isoforms have different biological properties depending on the presence or absence of exons 6 and 7, which encode the heparin-binding domain (HBD) involved in interactions with extracellular matrix proteins [12, 13]. Therefore, their effects on vascular endothelial cells vary [14]. Previous studies have recognized *VEGF-A121* and *VEGF-A165* as important isoforms in angiogenesis [15, 16]. However, differences in their biological functions have not yet been elucidated in detail. In recent years, *VEGF-A* isoform expression patterns have been shown to vary according to tumor type and microenvironment conditions [17], and correlations have been established between the dynamics of *VEGF-A* isoform expression in tumor tissues and patient prognosis [18–20]. These findings suggest that tumors adapt to their environment, including hypoxic and low-glucose environments, by changing the expression dynamics of *VEGF-A* isoforms [21–23]. However, there are no reports on changes in *VEGF-A* isoforms under hypoxic and low-glucose conditions.

Previously, we independently developed a new enzyme-linked immunosorbent assay (ELISA) system capable of separately detecting human VEGF-A121 and VEGF-A165 [24]. In the present study, to examine the relationship between metabolic stress conditions in the tumor microenvironment (specifically hypoxia and reduced glucose availability) and angiogenesis-related endothelial responses, we cultured human liver cancer-derived cell lines under hypoxic and low-glucose conditions and analyzed the expression patterns of VEGF-A isoforms using this original ELISA system.

## Methods

### Cells

The hepatocellular carcinoma cell line HepG2 was purchased from the American Type Culture Collection (ATCC, Manassas, VA). HepG2 cells were cultured in high-glucose Dulbecco’s Modified Eagle Medium (DMEM; Thermo Fisher Scientific, Waltham, MA) supplemented with 10% fetal bovine serum (FBS) and incubated at 37 °C in the presence of 5% CO_2_ for 24 h as a pre-culture. After pre-culture, the medium was replaced with high- or low-glucose DMEM (0.1 g/L D-glucose; Thermo Fisher Scientific) supplemented with 10% FBS, and cells were cultured under hypoxic conditions (< 1.0% O_2_, 5% CO_2_). For the low-glucose condition, osmolarity was adjusted by adding mannitol. The AMP-activated protein kinase (AMPK) activator A769662 (Abcam, #ab120335, Cambridge, MA), the AMPK inhibitor Compound C (Selleck, #S7306, Houston, TX), and the p38 mitogen-activated protein kinase (p38 MAPK) inhibitor SB203580 (Selleck, #S1076) were added 30 min before hypoxic stimulation.

Human umbilical vein endothelial cells (HUVECs) were purchased from Lonza (Basel, Switzerland) and maintained in fully supplemented endothelial growth medium-2 (EGM-2; Lonza) at 37 °C in a 5% CO_2_ incubator. For experiments, HUVECs were cultured overnight in EGM-2, after which the medium was replaced with endothelial basal medium-2 (EBM-2; Lonza) supplemented with 0.2% bovine serum albumin (BSA) (Sigma-Aldrich, St. Louis, MO) and incubated for 4 h. For the 3-(4,5-dimethyl-2-thiazolyl)−2,5-diphenyl-2H-tetrazolium bromide (MTT) assay, cells were seeded in 96-well plates at a density of 1 × 10^4^ cells/well. For the scratch assay and western blot analysis, cells were seeded in 12-well plates at a density of 1 × 10^5^ cells/well.

### Quantitative reverse transcription polymerase chain reaction

Total RNA was extracted from HepG2 cells using the RNeasy Mini Kit (Qiagen, Venlo, The Netherlands) according to the manufacturer’s protocol and reverse-transcribed into cDNA using the High-Capacity cDNA Reverse Transcription Kit (Roche Diagnostics, Basel, Switzerland). Quantitative reverse transcription polymerase chain reaction was performed to measure relative mRNA levels using Power SYBR Green PCR Master Mix and TaqMan™ Gene Expression Master Mix (Applied Biosystems, Foster City, CA). *VEGF-A121* and *VEGF-A165* expression was measured using the Power SYBR Green PCR Master Mix. The primer sequences were as follows: 5′-TGAGGAGTCCAACATCACCA-3′ and 5′-CTTGTCACATTTTTCTTGTC-3′ for *VEGF-A121* and 5′-CCCTGATGAGATCGAGTACATCTTCAAGC-3′ and 5′-AGCAAGGCCCACAGGGATTT-3′ for *VEGF-A165*. *VEGF-A* and *ACTB* expression was measured using TaqMan™ Gene Expression Master Mix and primers (*VEGF-A* # Hs00900055_m1 and *ACTB* #Hs01060665_g1). The samples were normalized to *ACTB* expression.

### ELISA

Both culture supernatants and whole-cell lysates were collected. Secreted VEGF-A121 and VEGF-A165 proteins in the supernatants and cell lysates were measured by ELISA as described previously [24]. Because cell growth differed depending on glucose concentration, total protein content was measured in the corresponding cell lysates, and VEGF levels in both supernatants and lysates were normalized to total cellular protein.

### Western blot analysis

Cells were lysed using lysis buffer (Cell Signaling Technology, Danvers, MA). Lysates were separated on 15% polyacrylamide gels (Bio-Rad, Hercules, CA) by sodium dodecyl-sulfate polyacrylamide gel electrophoresis and transferred to nitrocellulose membrane (Bio-Rad) at 400 mA for 35 min. Membranes were blocked in phosphate-buffered saline containing Tween 20 and 5% BSA, then incubated overnight at 4 °C with primary antibodies, followed by incubation for 1 h with a horseradish peroxidase-conjugated secondary antibody. Protein bands were visualized using SuperSignal Western Blot Enhancer (Thermo Fisher Scientific) and Amersham IQ500 (Cytiva, Marlborough, MA). Anti-HIF-1α antibodies were obtained from BD Transduction Laboratories (Franklin Lakes, NJ). Anti-HIF-2α, anti-AMPK, anti-phospho-AMPK, anti-p38 MAPK, anti-phospho-p38 MAPK, anti-Akt, anti-phospho-Akt, anti-p44/42, and anti-phospho-p44/42 antibodies were obtained from Cell Signaling Technology, while anti-actin antibodies and anti-GAPDH antibodies were obtained from Santa Cruz Biotechnology (Dallas, TX).

### MTT assay

After culturing HUVECs in EBM-2 medium containing 0.2% BSA and 0.5% FBS for 4 h, recombinant VEGF-A121, recombinant VEGF-A165 (Cell Signaling Technology), and the VEGF-A antibody bevacizumab (Chugai Pharmaceutical, Tokyo, Japan) were added, and cells were incubated for 24 h at 37℃ in the presence of 5% CO_2_. Then, 10 µL of MTT solution (5 mg/mL) was added and incubated for 4 h. The culture medium was then removed, and 100 µL of dimethyl sulfoxide was added to lyse the cells. Optical density was measured at 570 nm.

### Scratch assay

After culturing HUVECs in EBM-2 medium containing 0.2% BSA and 0.5% FBS for 4 h, the HUVEC monolayer was scratched along the center using a 1000-μL pipette tip. The medium was replaced with fresh EBM-2. Recombinant VEGF-A121, recombinant VEGF-A165, and the VEGF-A antibody bevacizumab were added, and cells were incubated at 37℃ in the presence of 5% CO_2_ for 15 h. Scratched areas were photographed before and after cell migration and compared.

### Tube formation assay

HUVECs were pre-incubated in EBM-2 medium containing 0.5% BSA for 4 h. Matrigel matrix (Corning Inc., Corning, NY) was dispensed into 96-well plates (50 µL/well) and allowed to polymerize at 37 °C for 30 min. HUVECs were then seeded onto the Matrigel at a density of 1 × 10^4^ cells/well in EBM-2 medium containing 0.5% BSA and 1% FBS and treated with recombinant VEGF-A121 (25 ng/mL; Cell Signaling Technology), recombinant VEGF-A165 (25 ng/mL; Cell Signaling Technology), and the anti-VEGF-A antibody bevacizumab (10 µg/mL; Chugai Pharmaceutical, Tokyo, Japan). After 8 h of incubation, tube-like structures were imaged using a BZ-X810 microscope (Keyence, Osaka, Japan). Tube formation parameters (total tube length, number of junctions, and number of meshes) were quantified using ImageJ with the Angiogenesis Analyzer plugin.

### Co-culture assay

Co-culture experiments were performed using HepG2 cells and HUVECs in a Transwell system (6.5-mm diameter, 0.4-µm pore size; Corning Costar, Corning Inc.). HepG2 cells were seeded onto Transwell inserts and cultured overnight in high-glucose DMEM supplemented with 10% FBS. The next day, the medium was replaced with EBM-2 containing 0.5% BSA under either high-glucose (4.5 g/L) or low-glucose (1.0 g/L) conditions; for the low-glucose condition, mannitol was added to match the osmolality of the high-glucose medium. Deferoxamine mesylate (DFX; Sigma-Aldrich) was added, and HepG2 cells were cultured for 18 h. HUVECs were seeded into 24-well plates and cultured overnight. The next day, the medium was replaced with EBM-2 containing 0.5% BSA under the same glucose and osmolality conditions, and cells were pretreated for 4 h. Subsequently, the Transwell inserts containing pretreated HepG2 cells were placed into wells containing HUVECs to initiate co-culture. DFX was also added to the HUVEC-containing wells at the start of co-culture. For western blot analysis, HUVECs were harvested 30 min after the initiation of co-culture. For the MTT assay, cell viability and proliferation were assessed 24 h after the initiation of co-culture.

### Statistical analysis

All experimental data are expressed as means ± standard deviation (SD) and were obtained from experiments repeated at least three times, unless otherwise indicated. Statistical analyses were performed using Student’s t-test, and *P*-values < 0.05 were considered statistically significant.

## Results

### Hypoxia and low-glucose conditions increase VEGF-A165 expression in HepG2 cells

**Fig. 1:**
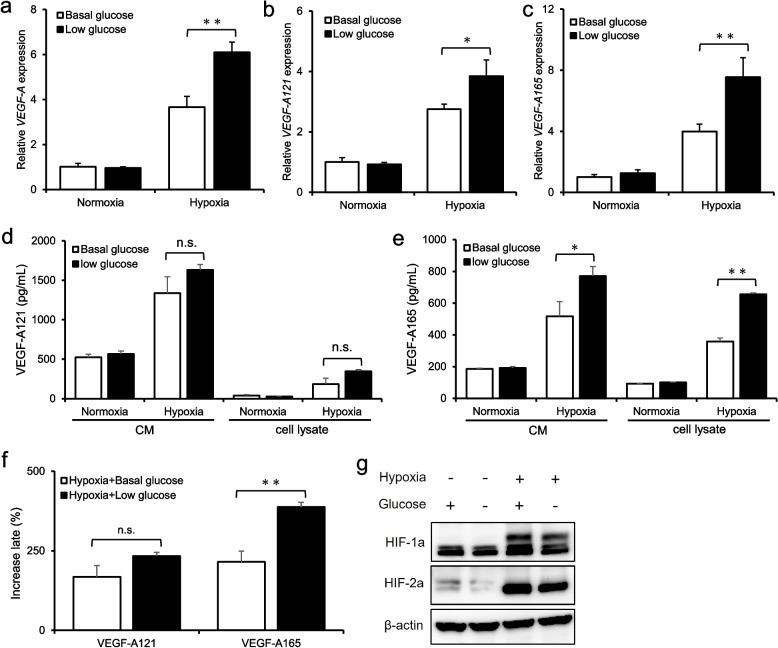
Hypoxia and low-glucose conditions increase *VEGF-A165* expression in HepG2 cells. **a**-**c** mRNA expression levels of *VEGF-A* (**a**), *VEGF-A121* (**b**), and *VEGF-A165* (**c**) at each glucose concentration (4.5 g/L or 0.1 g/L) under hypoxic conditions (< 1.0% O_2_) for 18 h (*n* = 4, mean ± SD; *P < 0.05, **P < 0.01). **d**-**e** Protein expression levels of VEGF-A121 (**d**) and VEGF-A165 (**e**) in conditioned medium and cell lysates at each glucose concentration (4.5 g/L or 0.1 g/L) under hypoxic conditions (< 1.0% O_2_) for 24 h. **f** Increase rate due to hypoxic stimulation (< 1.0% O_2_) at each glucose concentration (4.5 g/L or 0.1 g/L). Data were calculated from the results of (**d**) and (**e**) (*n* = 3, mean ± SD; ns, not significant; *P < 0.05, **P < 0.01). **g** Protein expression levels in cell lysates at each glucose concentration (4.5 g/L or 0.1 g/L) under hypoxic conditions (< 1.0% O_2_) for 6 h. Anti-HIF-1α, anti-HIF-2α, and anti-β-actin antibodies were used

Hypoxia and reduced glucose availability are major features of the tumor microenvironment. Previous studies have shown that VEGF-A expression is upregulated in HepG2 cells exposed to hypoxic or low-glucose conditions [21, 23]. To determine whether VEGF-A mRNA expression is increased by hypoxia and low-glucose stress, HepG2 cells were cultured under hypoxic conditions for 18 h at normal (4.5 g/L) or low (0.1 g/L) glucose concentrations. VEGF-A (Fig. 1a), VEGF-A121 (Fig. 1b), and VEGF-A165 (Fig. 1c) mRNA expression increased at low glucose concentrations compared to normal glucose concentrations under hypoxic conditions.

Next, we investigated the protein expression of VEGF-A isoforms in conditioned medium and cell lysates. VEGF-A165 protein expression increased after 24 h under low-glucose conditions compared to normal glucose conditions during hypoxia (Fig. 1e); the increase in VEGF-A121 protein expression was not statistically significant (Fig. 1 d). Based on these results, we calculated the rate of increase induced by hypoxic stimulation. VEGF-A165 protein expression increased by 215% at normal glucose concentrations and by 387% at low glucose concentrations (Fig. 1f). These results suggest that limited glucose availability under hypoxic conditions selectively increases VEGF-A165 protein expression.

Since HIF-1α and HIF-2α are the main regulators of VEGF-A expression under hypoxic conditions in HepG2 cells [25], we investigated their involvement in the increased expression of VEGF-A165 observed under hypoxic and low-glucose conditions. There was no increase in HIF-1α or HIF-2α expression after 6 h under low-glucose conditions compared with normal-glucose conditions (Fig. 1 g). However, knockdown of HIF-1α and HIF-2α reduced the protein expression levels of both VEGF-A121 and VEGF-A165 (Additional file 1). Taken together, these results suggest that the increase in VEGF-A165 protein expression under low-glucose conditions is not solely attributable to changes in HIF protein levels; nevertheless, HIF proteins are indispensable for VEGF-A121 and VEGF-A165 expression under these conditions.

### AMPK regulates VEGF-A165 expression under hypoxic and low-glucose conditions in HepG2 cells

**Fig. 2:**
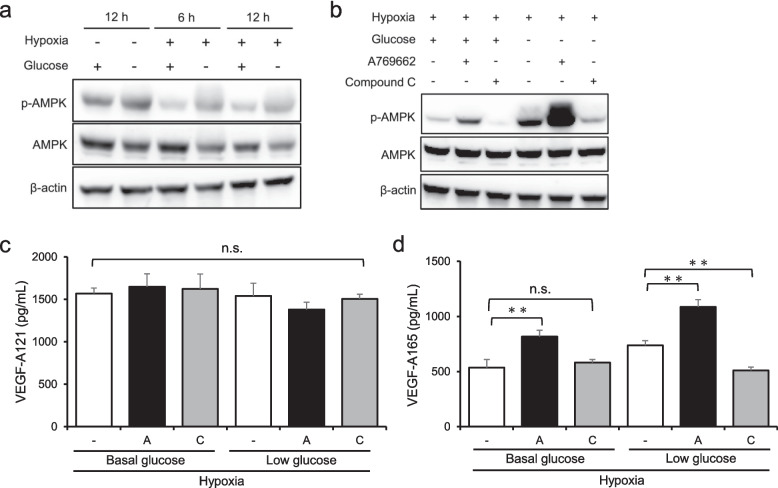
AMPK regulates VEGF-A165 expression under hypoxic and low-glucose conditions in HepG2 cells. **a** Protein expression levels in cell lysates at each glucose concentration (4.5 g/L or 0.1 g/L) under hypoxic conditions (< 1.0% O_2_) for 6 and 12 h. **b** Protein expression levels in cell lysates with A769662 (A, 100 μM), Compound C (C, 3 μM), and untreated control (−) at each glucose concentration (4.5 g/L or 0.1 g/L) under hypoxic conditions (< 1.0% O_2_) for 12 h. Anti-AMPK, anti- phosphorylated activated AMPK, and anti-β-actin antibodies were used in (**a**) and (**b**). **c** and **d**: Protein expression levels of VEGF-A121 (**c**) and VEGF-A165 (**d**) in conditioned medium with A769662 (A, 100 μM), Compound C (C, 3 μM), and untreated control (−) at each glucose concentration (4.5 g/L or 0.1 g/L) under hypoxic conditions (< 1.0% O_2_) for 24 h. (*n* = 3 ~ 4, mean ± SD; ns, not significant; **P < 0.01)

Previous studies have shown that the stability of *VEGF-A* mRNA is enhanced through AMPK/p38 MAPK signaling under low-glucose conditions [26]; therefore, we investigated the role of AMPK/p38 MAPK signaling in regulating the expression of VEGF-A165. Hypoxia slightly increased AMPK phosphorylation at Thr172 compared to normoxia, whereas low-glucose stimulation dramatically increased AMPK phosphorylation after 12 h (Fig. 2a). Treatment with the AMPK activator A769662 increased AMPK phosphorylation at normal glucose concentrations and further increased phosphorylation at low glucose concentrations under hypoxic conditions for 12 h (Fig. 2b). In contrast, treatment with the AMPK inhibitor Compound C reduced AMPK phosphorylation at normal and low glucose concentrations under hypoxic conditions for 12 h (Fig. 2b). A769662 increased VEGF-A165 protein expression at normal glucose concentrations and further enhanced expression at low glucose concentrations (Fig. 2d) but did not increase VEGF-A121 protein expression (Fig. 2c). In contrast, Compound C suppressed the increase in VEGF-A165 protein expression at low glucose concentrations (Fig. 2d) but had no effect at normal glucose concentrations (Fig. 2d). There was no change in VEGF-A121 levels at either normal or low glucose concentrations (Fig. 2c). These results suggest that AMPK activation contributes to the increased VEGF-A165 protein expression observed at low-glucose conditions.

### AMPK/p38 MAPK signaling regulates VEGF-A165 expression under hypoxia and low-glucose conditions in HepG2 cells

**Fig. 3:**
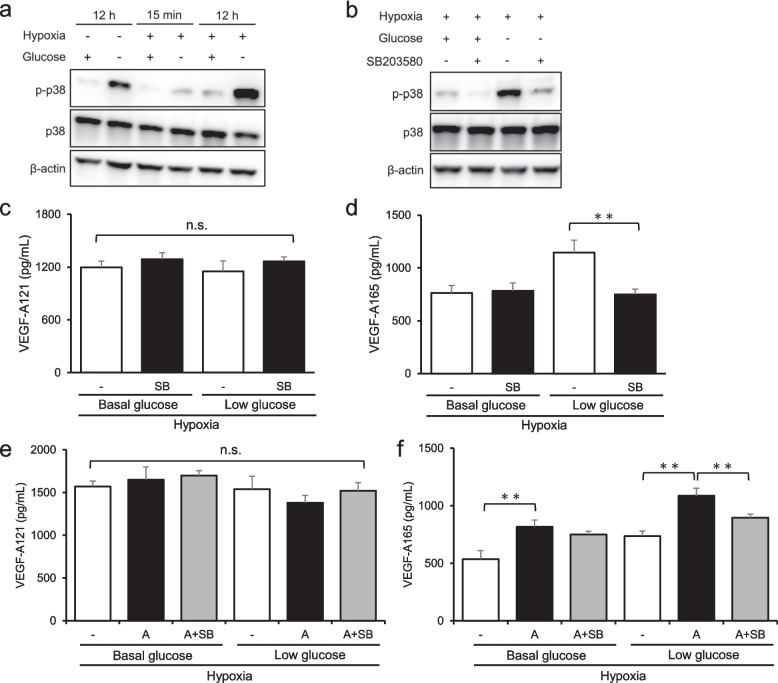
AMPK/p38 MAPK signaling regulates VEGF-A165 expression under hypoxia and low-glucose conditions in HepG2 cells. **a** Protein expression levels in cell lysates at each glucose concentration (4.5 g/L or 0.1 g/L) under hypoxic conditions (< 1.0% O_2_) at 15 min and 12 h. **b** Protein expression levels in cell lysates with SB203580 (SB, 10 μM) and untreated control (−) at each glucose concentration (4.5 g/L or 0.1 g/L) under hypoxic conditions (< 1.0% O_2_) for 15 min. Anti-p38 MAPK, anti-phosphorylated activated p38 MAPK, and anti-β-actin antibodies were used in (**a**) and (**b**). **c** and **d**: Protein expression levels of VEGF-A121 (**c**) and VEGF-A165 (**d**) in conditioned medium with SB203580 (SB, 10 μM) and untreated control (−) at each glucose concentration (4.5 g/L or 0.1 g/L) under hypoxic conditions (< 1.0% O_2_) for 24 h. **e** and **f**: Protein expression levels of VEGF-A121 (**e**) and VEGF-A165 (**f**) in conditioned medium with only A769662 (A, 100 μM) or A769662 and SB203580 (A + SB, 100 μM + 10 μM) and untreated control (−) at each glucose concentration (4.5 g/L or 0.1 g/L) under hypoxic conditions (< 1.0% O_2_) for 24 h. (*n* = 4, mean ± SD; ns, not significant; **P < 0.01)

Next, we investigated whether p38 MAPK is involved in the increased expression of VEGF-A165 under hypoxic and low-glucose conditions. Hypoxia slightly increased p38 MAPK phosphorylation at Thr180/Tyr182 compared to normoxia, whereas low-glucose stimulation dramatically increased p38 MAPK phosphorylation after 12 h (Fig. 3a). Under hypoxic conditions, low glucose increased p38 MAPK phosphorylation after 15 min, with a further dramatic increase after 12 h (Fig. 3a). Treatment with the p38 MAPK inhibitor SB203580 reduced p38 MAPK phosphorylation at normal and low glucose concentrations under hypoxia for 15 min (Fig. 3b). SB203580 suppressed low glucose-induced VEGF-A165 protein expression; however, no change was observed at normal glucose concentrations (Fig. 3d). There was no change in VEGF-A121 protein levels at either normal or low glucose concentrations (Fig. 3c). Treatment with A769662 and SB203580 inhibited the A769662-induced increase in VEGF-A165 protein expression at low glucose concentrations; no change was observed at normal glucose concentrations (Fig. 3f). There was no change in VEGF-A121 expression after treatment with A769662 or SB203580 at either normal or low glucose concentrations (Fig. 3e). These results suggest that AMPK/p38 MAPK signaling is involved in increasing VEGF-A165 protein expression under low-glucose conditions.

### VEGF-A165 promotes endothelial cell proliferation and migration more than VEGF-A121 in HUVECs

**Fig. 4:**
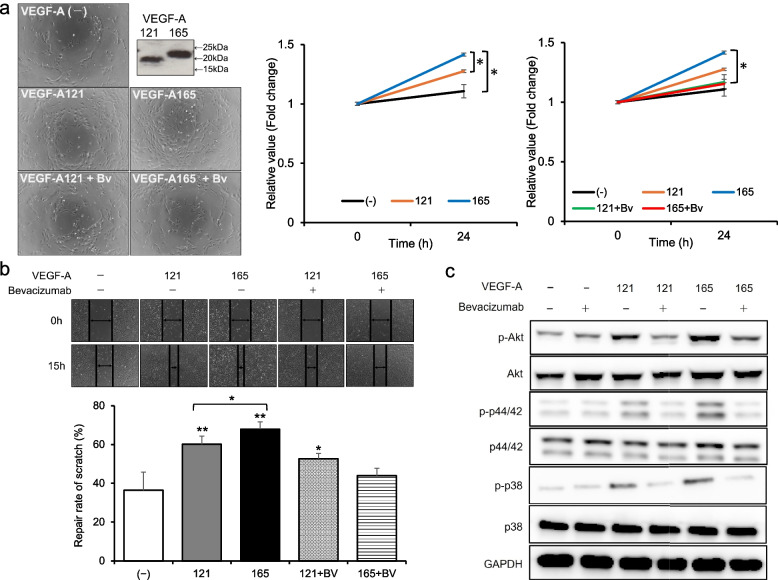
VEGF-A165 promotes endothelial cell proliferation and migration more than VEGF-A121 in HUVECs. **a** MTT assay using HUVECs cultured for 24 h (*n* = 3, mean ± SD; *P < 0.05). **b** Scratch assay using HUVECs cultured for 15 h (*n* = 4, mean ± SD; *P < 0.05, **P < 0.01). **c** Protein expression levels in cell lysates of HUVECs incubated for 30 min. Anti-Akt, anti-phosphorylated activated Akt anti-p38 MAPK, anti-phosphorylated activated p38 MAPK anti-p44/42 MAPK, anti-phosphorylated activated p44/42 MAPK, and anti-GAPDH antibodies were used. In (**a**) and (**c**), HUVECs were treated with VEGF-A121 and VEGF-A165 recombinant proteins (25 ng/mL) and bevacizumab (10 µg/mL), whereas in (**b**), HUVECs were treated with VEGF-A121 and VEGF-A165 recombinant proteins (0.65 nM) and bevacizumab (10 µg/mL)

To compare the effects of VEGF-A isoforms on endothelial cell responses related to angiogenesis, we investigated their effects on HUVECs using recombinant VEGF-A121 and VEGF-A165. Previous studies have shown that the proliferation, migration, and tube formation of vascular endothelial cells are important for angiogenesis [27]. VEGF-A165 enhanced HUVEC proliferation more strongly than VEGF-A121, and this effect was suppressed by the addition of bevacizumab, an anti-pan VEGF-A antibody (Fig. 4a). Similarly, VEGF-A165 enhanced HUVEC migration ability more effectively than VEGF-A121, and this effect was also suppressed by bevacizumab (Fig. 4b). Both VEGF-A121 and VEGF-A165 promoted tube formation, and this effect was suppressed by bevacizumab; however, total tube length was slightly but significantly greater with VEGF-A165 than with VEGF-A121 (Additional file 2). Because signaling molecules such as Akt, p38 MAPK, and p44/42 MAPK are involved in the proliferation and migration of HUVECs [28], we assessed their phosphorylation status by western blot analysis. VEGF-A165 promoted the phosphorylation of Akt, p38 MAPK, and p44/42 MAPK more strongly than VEGF-A121, and these effects were suppressed by bevacizumab (Fig. 4c).

Finally, we examined angiogenic signaling in a tumor–endothelial interaction model using a co-culture system of HepG2 cells and HUVECs. In the MTT assay, co-culture under low-glucose conditions significantly increased HUVEC viability and proliferation compared with co-culture under high-glucose conditions (Additional file 3). Western blot analysis showed that phosphorylation of Akt, p38 MAPK, and p44/42 MAPK in HUVECs was enhanced by co-culture with HepG2 and was further increased under low-glucose conditions compared with high-glucose conditions (Additional file 3). Overall, signaling and functional analyses using recombinant VEGF indicated that VEGF-A165 more strongly promotes angiogenesis-related responses than VEGF-A121, and the co-culture experiments yielded consistent results.

## Discussion

Tumor cells grow rapidly and require large amounts of oxygen, which exposes them to a hypoxic environment [1]. To adapt, they activate glycolysis to produce ATP [3, 4] and increase VEGF-A expression to promote angiogenesis [6]. These behaviors are controlled by HIF-1α in a hypoxic environment. Previous studies have confirmed that VEGF-A expression also increases when glucose, the main energy source for tumor cells, is limited. In human hepatocellular carcinoma cell lines, *VEGF-A* mRNA transcription is reportedly upregulated via unfolded protein response activation [22] and the DNA binding activity of the transcription factor AP1 [29], both of which are HIF-independent. However, it is unclear how the expression of VEGF-A and its isoforms changes under low-glucose hypoxic conditions that closely resemble the actual tumor microenvironment.

In this study, we found that restricting glucose under hypoxia increased VEGF-A165 expression through the cooperative effect of HIF proteins and additional regulatory mechanisms (Fig. 1). To assess generalizability beyond HepG2 cells, we examined additional tumor cell lines with relatively high VEGF-A expression, including Huh7 (human hepatocellular carcinoma), HCT116 (human colorectal carcinoma), and A549 (human lung adenocarcinoma). Huh7 cells showed a similar increase in VEGF-A165 under combined hypoxic and low-glucose conditions, whereas HCT116 cells tended to exhibit reduced overall VEGF-A protein levels under low glucose (Additional file 4). In A549 cells, low glucose markedly reduced viability, precluding reliable quantification of VEGF-A under our experimental conditions (data not shown). Collectively, these observations suggest that the low-glucose–associated increase in VEGF-A165 is cell type- and context-dependent and may be particularly prominent in hepatocellular carcinoma cells, which exhibit distinctive metabolic programs [30].

Regarding HIF-dependent transcriptional regulation, it was recently reported that recruitment of specific transcription factors to hypoxia response elements can further augment the expression of HIF target genes [31]. Accordingly, we assessed VEGF-A165 expression by knocking out candidate transcription factors whose activity increased under low-glucose conditions; however, we did not identify any factors that upregulated VEGF-A165 under hypoxia and low glucose (Additional file 5).

By contrast, as an HIF-independent regulatory mechanism, AMPK/p38 MAPK signaling has been reported to enhance *VEGF-A* mRNA stability under low-glucose conditions [26]. Consistent with this, our results suggest involvement of AMPK/p38 MAPK signaling (Figs. 2, and 3), raising the possibility that AMPK/p38 acts at post-transcriptional stages on HIF-induced *VEGF-A165* mRNA, thereby increasing VEGF-A165 protein expression. However, although inhibition of AMPK or p38 reduced *VEGF-A165* mRNA levels, AMPK activation did not increase *VEGF-A165* mRNA; thus, enhanced mRNA stability could not be directly demonstrated in the present study (Additional file 6). Therefore, AMPK/p38 signaling may contribute not only to mRNA stability but also to other post-transcriptional regulatory processes. Because VEGF-A isoforms are generated by alternative splicing of the *VEGF-A* gene [11], their regulation may involve multiple layers of control, including modulation of splicing balance. In addition, regulation at the levels of translation, post-translational modification, and miRNA-mediated mechanisms has been reported [32]. Further systematic studies are required to clarify which regulatory steps are targeted by AMPK/p38 signaling to drive the selective increase in VEGF-A165.

Next, we assessed the biological functions of the VEGF-A isoforms. These isoforms have different biological properties depending on the presence of exons 6 and 7, which encode the HBD involved in interactions with extracellular matrix proteins [12, 13]. VEGF-A121, which lacks exons 6 and 7, is present in the blood, while VEGF-A165, which contains only exon 6, is present in both the blood and tissue stroma [13]. These differences are known to affect biological functions such as vascular maturation, including vessel caliber, density, and permeability [17]. Our study confirmed that VEGF-A165 more effectively activates vascular endothelial cell function (Fig. 4), whereas VEGF-A121 is present at higher concentrations than VEGF-A165 in HepG2-conditioned medium (CM). Therefore, the angiogenic activity observed as the total CM activity likely mainly reflects the quantitative contribution of VEGF-A121. However, VEGF-A165, via its HBD, preferentially interacts with extracellular matrix components and neuropilin-1, and its retention within tissues may generate perivascular concentration gradients that more efficiently activate VEGFR2 signaling [33]. Thus, assessing isoform contribution solely based on the total activity of CM has inherent limitations, and extrapolation to the tumor microenvironment should consider not only isoform abundance but also differences in localization and bioavailability.

A key limitation of this study is the lack of tube formation assays using CM from cancer cells cultured under hypoxia and low glucose. Accordingly, our functional data primarily support enhanced endothelial proliferation and migration, rather than angiogenesis per se. Future studies using CM from tumor cells exposed to hypoxia and reduced glucose are needed to directly demonstrate functional vascular network formation and to strengthen physiological relevance.

Collectively, these findings suggest that a selective increase in VEGF-A165 under metabolic stress, such as hypoxia and low glucose, may represent a strategy to enhance pro-angiogenic endothelial responses in situ even when energy resources are limited. Moreover, tumor cells may adjust the expression ratio and spatial distribution of VEGF-A isoforms in response to environmental cues to create a microenvironment favorable for survival and proliferation.

There have been many reports on the relationship between VEGF-A isoform expression and tumor behavior in vivo. For example, mice expressing VEGF-A165 exhibit faster tumor growth than those expressing VEGF-A121 [34]. In addition, in tumors such as hepatocellular carcinoma, colon cancer, and glioblastoma, the expression levels and ratios of VEGF-A isoforms correlate with tumor severity, prognosis, and recurrence [18, 19, 35]. The measurement of VEGF-A isoforms has also been reported to predict clinical outcomes in gastric and pancreatic cancers after treatment with bevacizumab [36, 37]. Furthermore, ongoing discussions are considering whether a possible link exists between tumor progression, prognosis, and VEGF-A isoforms. These findings highlight the clinical importance of assessing VEGF-A isoform expression for determining tumor pathology, treatment, and prognosis; however, a specific measurement system for VEGF-A isoforms has not yet been developed. Using our specific VEGF-A isoform measurement system, we demonstrated for the first time that VEGF-A165 protein expression selectively increases in HepG2 cells when glucose is restricted under hypoxia. In the future, this measurement system may be used to clarify the expression dynamics, significance, and control mechanisms of VEGF-A isoforms in diverse tumor microenvironments. It is hoped that VEGF-A isoforms will be established as biomarkers for tumor pathology, prognosis, and treatment response.

## Conclusion

In this study, we demonstrated for the first time that VEGF-A165 protein expression is selectively increased in HepG2 cells under hypoxic and low-glucose conditions via HIF and AMPK/p38 MAPK signaling. Because VEGF-A165 exerts stronger effects on vascular endothelial cells than VEGF-A121, its expression may be selectively upregulated under stress conditions to promote endothelial cell proliferation and migration. These findings suggest that tumors adapt to their environment by altering the expression dynamics of VEGF-A isoforms.

## Supplementary Information

Additional file 1. Protein expression levels of VEGF-A165 in conditioned medium in HepG2 cells transfected with silencing RNA targeting HIF-1α and HIF-2α (SiHIF) or control (SiC) at each glucose concentration (4.5 g/L or 0.1 g/L) under hypoxic conditions (<1.0% O_2_) for 24 h. (*n*=3, mean ± SD; **P<0.01). Additional file 2. Representative phase-contrast images (upper left) of capillary-like structures formed by HUVECs cultured without VEGF-A [VEGF-A (−)], with VEGF-A121, with VEGF-A165, or with each VEGF-A isoform in the presence of bevacizumab (BV). Quantitative analyses of total tube length (a), number of junctions (b), and number of meshes (c) are shown. (*n*=4~6, mean ± SD; ns, not significant; *P < 0.05, **P<0.01). Additional file 3. (a): MTT assay in HUVECs after 24 h. Data are shown as absorbance at 570 nm (OD_570) under high-glucose (H) and low-glucose (L) conditions in the presence of HepG2 co-culture [HepG2 (+)] (*n*=6, mean ± SD; **P < 0.01). (b): Protein expression levels in cell lysates of HUVECs incubated for 30 min. In (a), and (b), HUVECs were co-cultured with HepG2 cells in a Transwell system, and DFX (100 µM) was added to the culture medium under either high-glucose (H) or low-glucose (L) conditions. Additional file 4. Protein expression levels of VEGF-A121 and VEGF-A165 in conditioned medium in Huh7 cells and HCT116 cells at each glucose concentration under hypoxic conditions (<1.0% O_2_) for 24 h (Huh7: 4.5 g/L or 0.1 g/L; HCT116: 4.5 g/L or 0.5 g/L). (*n*=2~3, mean ± SD; ns, not significant; *P< 0.05, **P < 0.01). Additional file 5. Protein expression levels of VEGF-A165 in conditioned medium in HepG2 cells transfected with silencing RNA of SP1 (SiSP1), EGR1 (SiEGR1), Nrf2 (SiNrf2), SIRT1 (SiSIRT1), PGC-1 (SiPGC-1) and control (SiC) at each glucose concentration (4.5 g/L or 0.1 g/L) under hypoxic conditions (<1.0% O_2_) for 24 h. (*n*=3, mean ± SD; ns, not significant; *P < 0.05, **P < 0.01). Additional file 6. (a): *VEGF-A165* mRNA expression under hypoxic conditions (<1.0% O_2_) at basal glucose concentration for 12 h in the presence of A769662 (A, 100 μM) or vehicle control (−) (*n*=3, mean ± SD; ns, not significan). (b): *VEGF-A165* mRNA expression under hypoxic conditions (<1.0% O_2_) at low glucose concentration for 12 h in the presence of A769662 (A, 100 μM), Compound C (C, 3 μM), SB203580 (SB, 10 μM) or vehicle control (−) (*n*=4, mean ± SD; ns, not significant; *P < 0.05, **P < 0.01).

## Data Availability

All data generated or analysed during this study are included in this published article and its supplementary information files.

## Abbreviations

AMPK: AMP-activated protein kinase
ATP: Adenosine triphosphate
BSA: Bovine serum albumin
DMEM: Dulbecco's Modified Eagle Medium
EBM-2: Endothelial basal medium-2
EGM-2: Endothelial growth medium-2
ELISA: Enzyme-linked immunosorbent assay
FBS: Fetal bovine serum
HBD: Heparin-binding domain
HIF-1α: Hypoxia-inducible factor-1α
HUVEC: Human umbilical vein endothelial cell
p38 MAPK: P38 mitogen-activated protein kinase
MTT: 3-(4,5-Dimethyl-2-thiazolyl)-2,5-diphenyl-2H-tetrazolium bromide
DFX: Deferoxamine mesylate
SD: Standard deviation
VEGF: Vascular endothelial growth factor
VEGF-A: Vascular endothelial growth factor-A
