# Supplementary material for: AMPK/p38 MAPK signaling selectively enhances HIF-induced VEGF-A165 expression under hypoxic and low-glucose conditions in HepG2 cells to promote endothelial cell proliferation and migration
Source: BMC Cancer. 2026 May 6;26:782. doi: 10.1186/s12885-026-16069-0 (PMC13317407; doi:10.1186/s12885-026-16069-0)
Supplement: Supplementary file 1 — Additional file 1. Protein expression levels of VEGF-A165 in conditioned medium in HepG2 cells transfected with silencing RNA targeting HIF-1α and HIF-2α (SiHIF) or control (SiC) at each glucose concentration (4.5 g/L or 0.1 g/L) under hypoxic conditions (<1.0% O2) for 24 h. (n=3, mean ± SD; **P<0.01). Additional file 2. Representative phase-contrast images (upper left) of capillary-like structures formed by HUVECs cultured without VEGF-A [VEGF-A (−)], with VEGF-A121, with VEGF-A165, or with each VEGF-A isoform in the presence of bevacizumab (BV). Quantitative analyses of total tube length (a), number of junctions (b), and number of meshes (c) are shown. (n=4~6, mean ± SD; ns, not significant; *P < 0.05, **P<0.01). Additional file 3. (a): MTT assay in HUVECs after 24 h. Data are shown as absorbance at 570 nm (OD_570) under high-glucose (H) and low-glucose (L) conditions in the presence of HepG2 co-culture [HepG2 (+)] (n=6, mean ± SD; **P < 0.01). (b): Protein expression levels in cell lysates of HUVECs incubated for 30 min. In (a), and (b), HUVECs were co-cultured with HepG2 cells in a Transwell system, and DFX (100 µM) was added to the culture medium under either high-glucose (H) or low-glucose (L) conditions. Additional file 4. Protein expression levels of VEGF-A121 and VEGF-A165 in conditioned medium in Huh7 cells and HCT116 cells at each glucose concentration under hypoxic conditions (<1.0% O2) for 24 h (Huh7: 4.5 g/L or 0.1 g/L; HCT116: 4.5 g/L or 0.5 g/L). (n=2~3, mean ± SD; ns, not significant; *P< 0.05, **P < 0.01). Additional file 5. Protein expression levels of VEGF-A165 in conditioned medium in HepG2 cells transfected with silencing RNA of SP1 (SiSP1), EGR1 (SiEGR1), Nrf2 (SiNrf2), SIRT1 (SiSIRT1), PGC-1 (SiPGC-1) and control (SiC) at each glucose concentration (4.5 g/L or 0.1 g/L) under hypoxic conditions (<1.0% O2) for 24 h. (n=3, mean ± SD; ns, not significant; *P < 0.05, **P < 0.01). Additional file 6. (a): VEGF-A165 mRNA expression under hypoxic cond [file 12885_2026_16069_MOESM1_ESM.zip › Additional file revised#3.docx]

**AMPK/p38 MAPK signaling selectively enhances HIF-induced VEGF-A165 expression under hypoxic and low-glucose conditions in HepG2 cells to promote endothelial cell proliferation and migration**

Hirohito Hashinokuchi^1,2^, Munekazu Yamakuchi^1,2*^, Sadayuki Higashi^1,2^, Kazunori Takenouchi^1,2^, Akito Tabaru^1^, Yoko Oyama^1,2^, Chieko Fujisaki^1,2^, Kiyonori Tanoue^1,2^, Masashi Okawa^3^, Fuminori Namino^2^, Teruto Hashiguchi^1,2*^

1. Department of Laboratory and Vascular Medicine, Graduate School of Medical and Dental Sciences, Kagoshima University, Kagoshima, Japan
2. Department of Laboratory and Medicine, Kagoshima University Hospital, Kagoshima, Japan
3. Department of Cardiovascular Surgery, Graduate School of Medical and Dental Sciences, Kagoshima University, Kagoshima, Japan

^*^Corresponding author: Munekazu Yamakuchi and Teruto Hashiguchi

E-mail address: [munekazu@m.kufm.kagoshima-u.ac.jp](mailto:munekazu@m.kufm.kagoshima-u.ac.jp) (MY), [k1581347@kadai.jp](mailto:k1581347@kadai.jp) (TH)

Address: Department of Laboratory and Vascular Medicine, Cardiovascular and Respiratory Disorders, Kagoshima University Graduate School of Medical and Dental Sciences, 8-35-1, Sakuragaoka, Kagoshima, 890-8520, Japan.

**
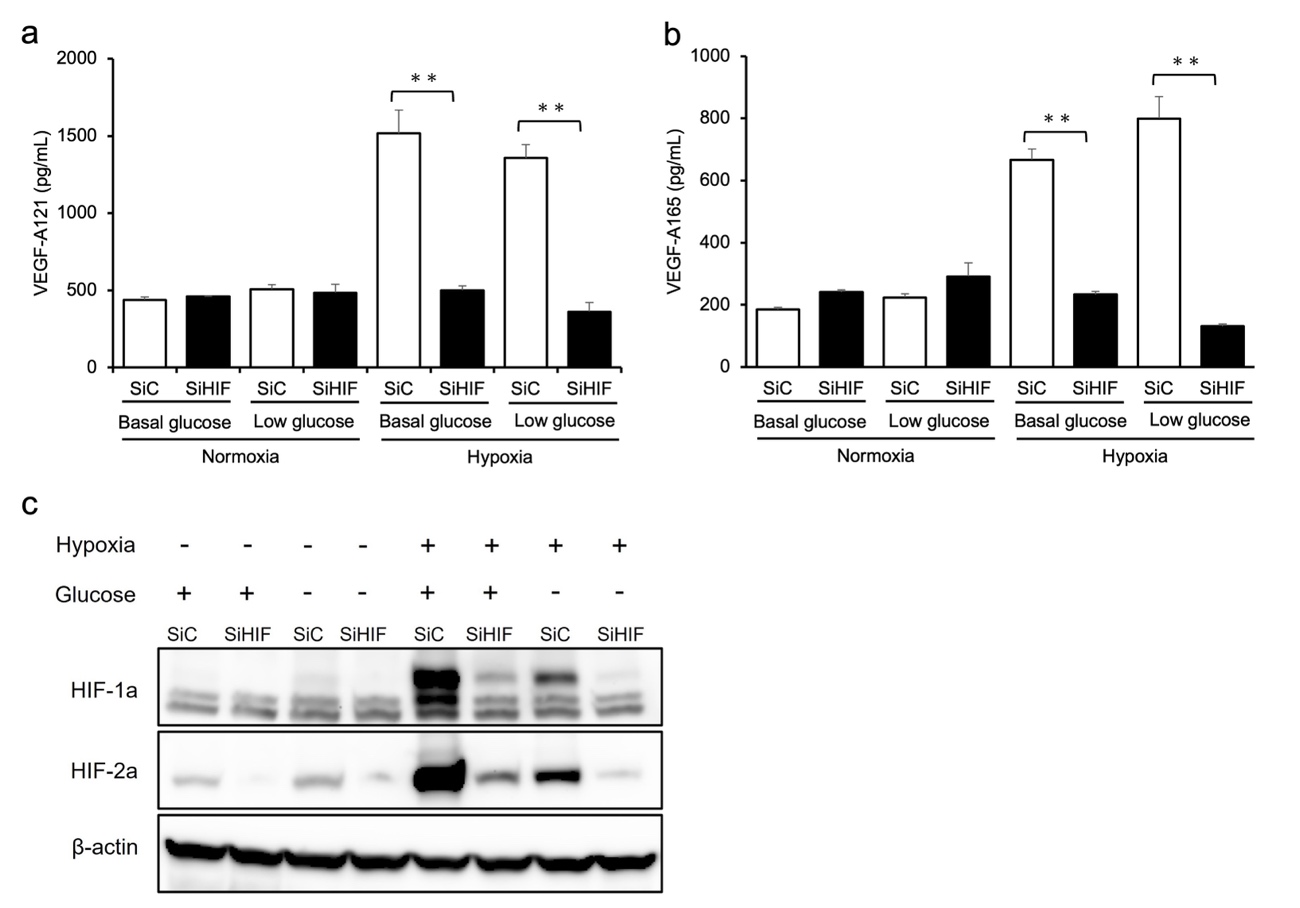
**

**Supplemental Fig. S1.** Protein expression levels of VEGF-A165 in conditioned medium in HepG2 cells transfected with silencing RNA targeting HIF-1α and HIF-2α (SiHIF) or control (SiC) at each glucose concentration (4.5 g/L or 0.1 g/L) under hypoxic conditions (0.5% O_2_) for 24 h. (n=3, mean ± SD; **P<0.01).


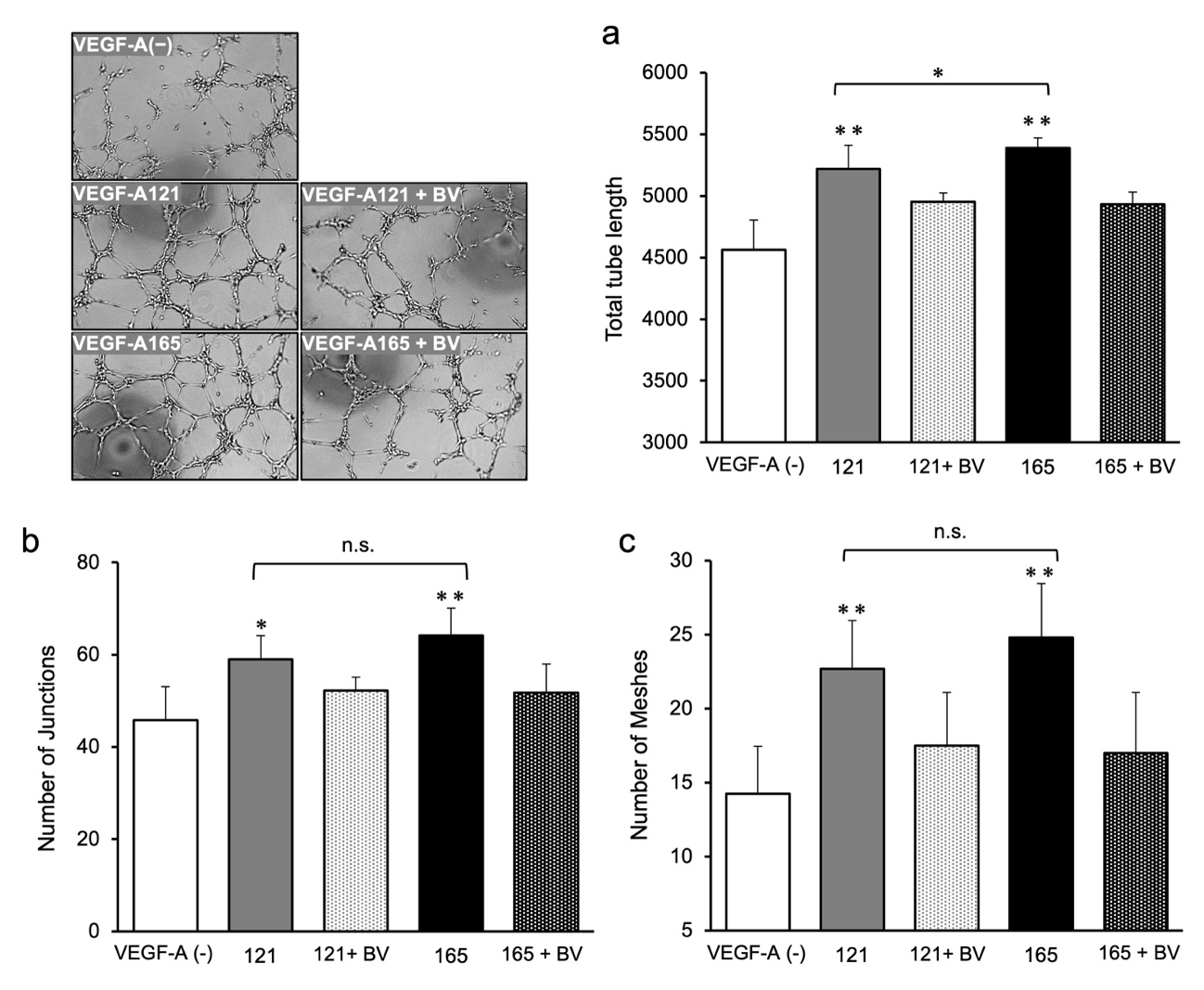


**Supplemental Fig. S2.** Representative phase-contrast images (upper right) of capillary-like structures formed by HUVECs cultured without VEGF-A [VEGF-A (−)], with VEGF-A121, with VEGF-A165, or with each VEGF-A isoform in the presence of bevacizumab (BV). Quantitative analyses of total tube length (a), number of junctions (b), and number of meshes (c) are shown. (n=4~6, mean ± SD; ns, not significant; *P < 0.05, **P<0.01)**.**


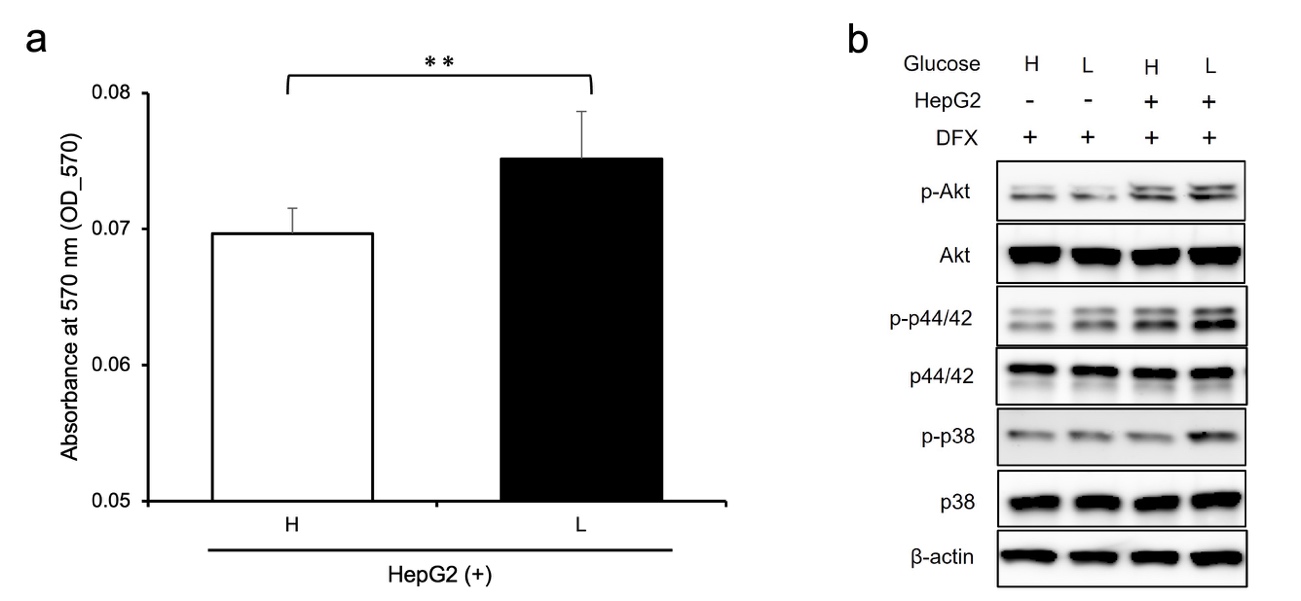


**Supplemental Fig. S3.** (a): MTT assay in HUVECs after 24 h. Data are shown as the low-glucose/high-glucose ratio of MTT absorbance in the absence [HepG2 (−)] or presence [HepG2 (+)] of HepG2 co-culture (n=6, mean ± SD; **P < 0.01). (b): Protein expression levels in cell lysates of HUVECs incubated for 30 min. In (a), and (b), HUVECs were co-cultured with HepG2 cells in a Transwell system, and DFX (100 µM) was added to the culture medium under either high-glucose (H) or low-glucose (L) conditions.


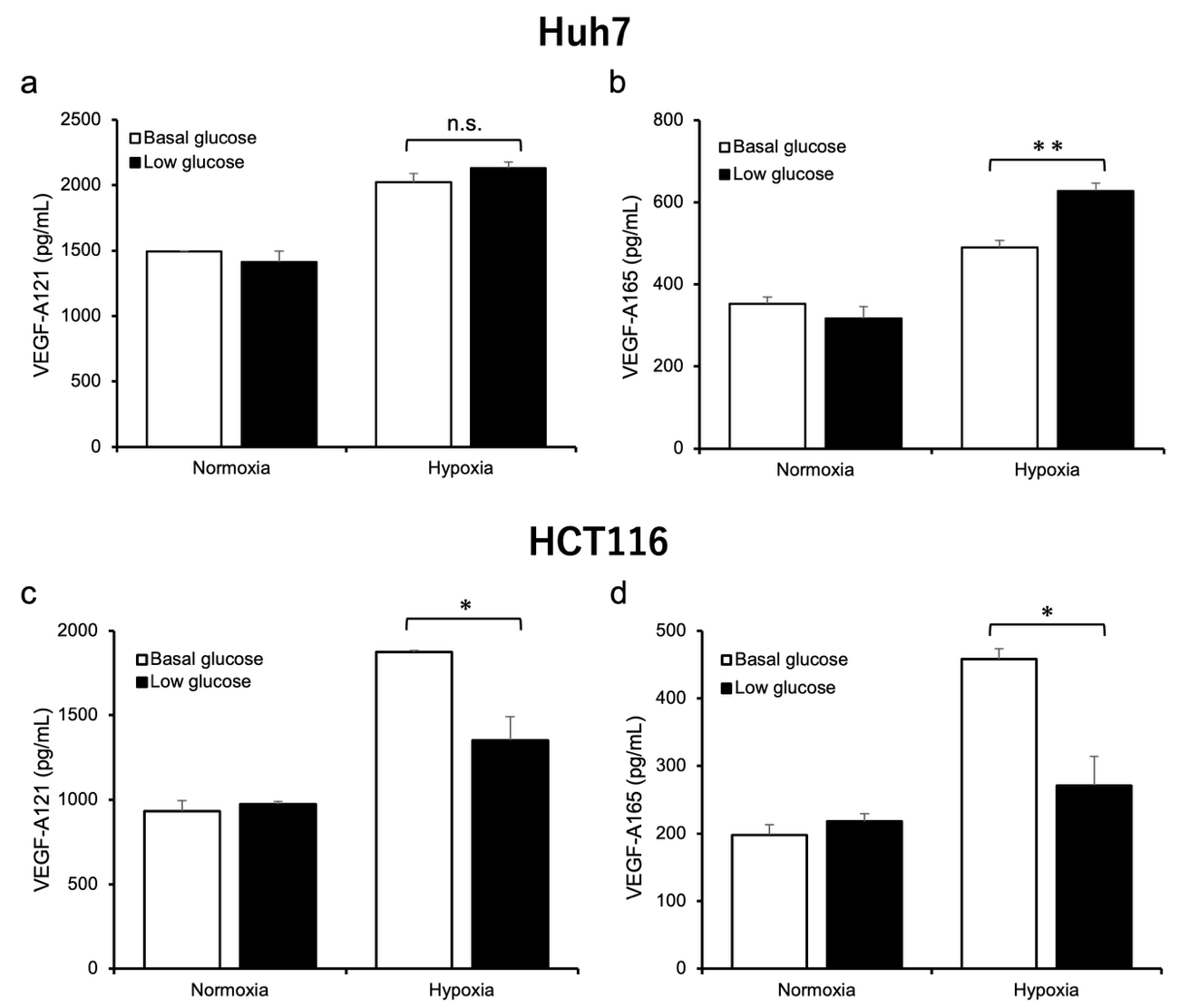


**Supplemental Fig. S4.** Protein expression levels of VEGF-A121 and VEGF-A165 in conditioned medium in Huh7 cells and HCT116 cells at each glucose concentration under hypoxic conditions (0.5% O2) for 24 h (Huh7: 4.5 g/L or 0.1 g/L; HCT116: 4.5 g/L or 0.5 g/L). (n=2~3, mean ± SD; ns, not significant; *P < 0.05, **P < 0.01).


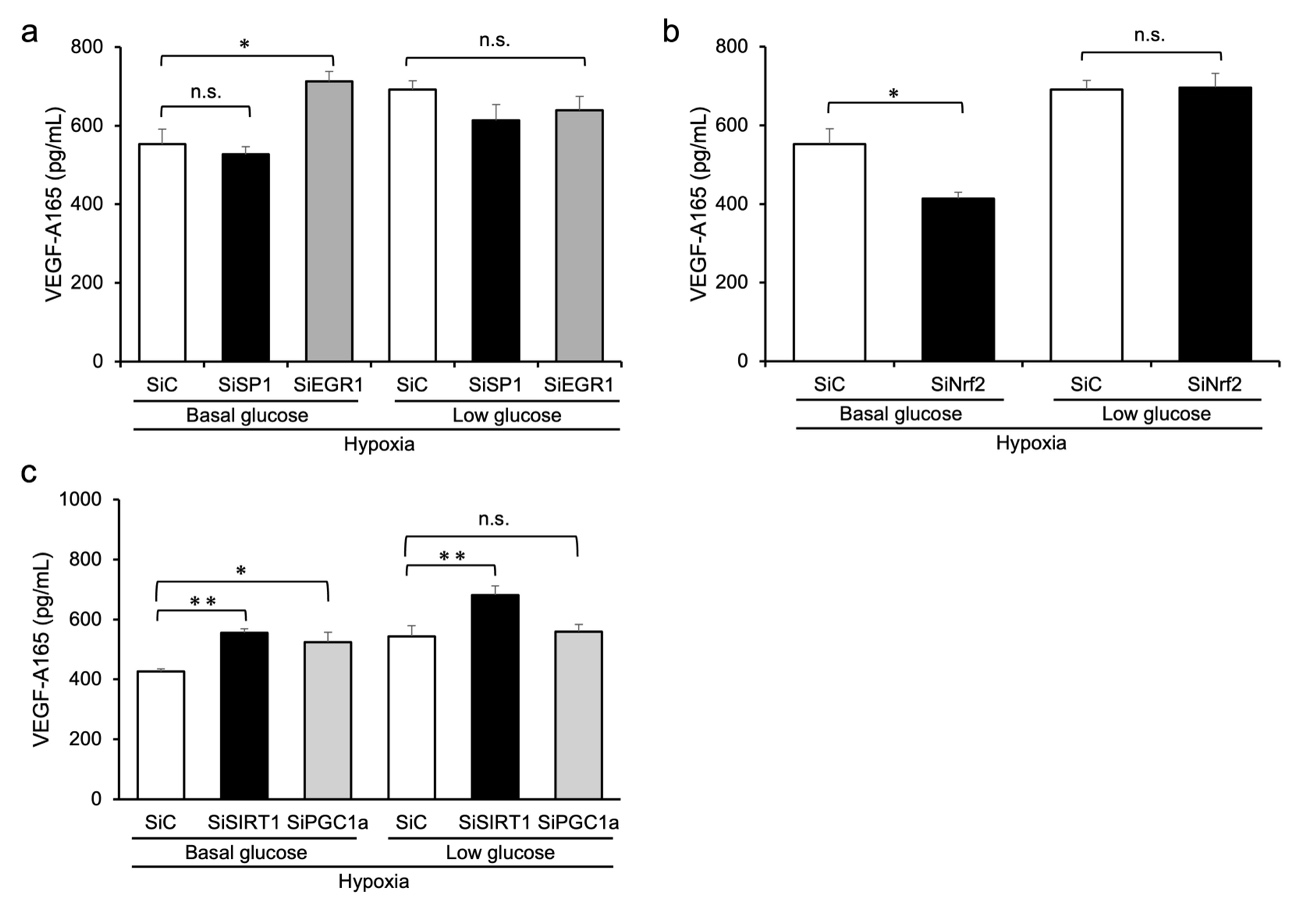


**Supplemental Fig. S5.** Protein expression levels of VEGF-A165 in conditioned medium in HepG2 cells transfected with silencing RNA of SP1 (SiSP1), EGR1 (SiEGR1), Nrf2 (SiNrf2), SIRT1 (SiSIRT1), PGC-1 (SiPGC-1) or control (SiC) at each glucose concentration (4.5 g/L or 0.1 g/L) under hypoxic conditions (0.5% O_2_) for 24 h. (n=3 ± SD; ns, not significant; *P < 0.05, **P<0.01).


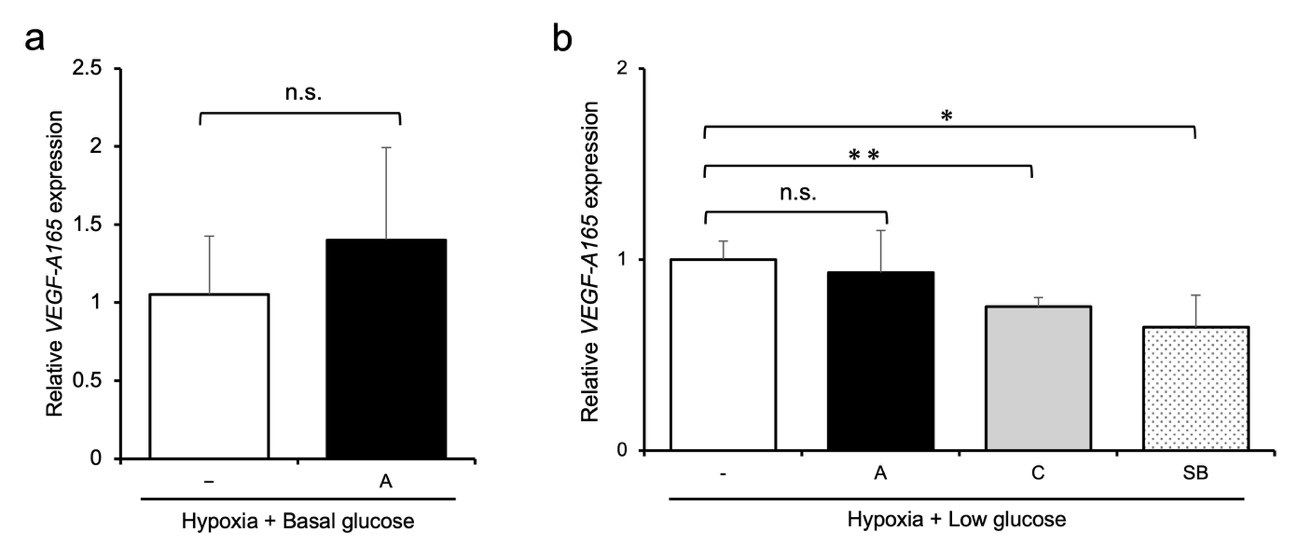


**Supplemental Fig. S6.** (a): *VEGF-A165* mRNA expression under hypoxic conditions (0.5% O_2_) at basal glucose concentration for 12 h in the presence of A769662 (A, 100 μM) or vehicle control (−) (n=3, mean ± SD; ns, not significant). (b): *VEGF-A165* mRNA expression under hypoxic conditions (0.5% O_2_) at low glucose concentration for 12 h in the presence of A769662 (A, 100 μM), Compound C (C, 3 μM), SB203580 (SB, 10 μM) or vehicle control (−). (n=4, mean ± SD; ns, not significant; *P < 0.05, **P < 0.01).
