# Supplementary material for: AMPK/p38 MAPK signaling selectively enhances HIF-induced VEGF-A165 expression under hypoxic and low-glucose conditions in HepG2 cells to promote endothelial cell proliferation and migration
Source: BMC Cancer. 2026 May 6;26:782. doi: 10.1186/s12885-026-16069-0 (PMC13317407; doi:10.1186/s12885-026-16069-0)
Supplement: Supplementary file 1 — Additional file 1. Protein expression levels of VEGF-A165 in conditioned medium in HepG2 cells transfected with silencing RNA targeting HIF-1α and HIF-2α (SiHIF) or control (SiC) at each glucose concentration (4.5 g/L or 0.1 g/L) under hypoxic conditions (<1.0% O2) for 24 h. (n=3, mean ± SD; **P<0.01). Additional file 2. Representative phase-contrast images (upper left) of capillary-like structures formed by HUVECs cultured without VEGF-A [VEGF-A (−)], with VEGF-A121, with VEGF-A165, or with each VEGF-A isoform in the presence of bevacizumab (BV). Quantitative analyses of total tube length (a), number of junctions (b), and number of meshes (c) are shown. (n=4~6, mean ± SD; ns, not significant; *P < 0.05, **P<0.01). Additional file 3. (a): MTT assay in HUVECs after 24 h. Data are shown as absorbance at 570 nm (OD_570) under high-glucose (H) and low-glucose (L) conditions in the presence of HepG2 co-culture [HepG2 (+)] (n=6, mean ± SD; **P < 0.01). (b): Protein expression levels in cell lysates of HUVECs incubated for 30 min. In (a), and (b), HUVECs were co-cultured with HepG2 cells in a Transwell system, and DFX (100 µM) was added to the culture medium under either high-glucose (H) or low-glucose (L) conditions. Additional file 4. Protein expression levels of VEGF-A121 and VEGF-A165 in conditioned medium in Huh7 cells and HCT116 cells at each glucose concentration under hypoxic conditions (<1.0% O2) for 24 h (Huh7: 4.5 g/L or 0.1 g/L; HCT116: 4.5 g/L or 0.5 g/L). (n=2~3, mean ± SD; ns, not significant; *P< 0.05, **P < 0.01). Additional file 5. Protein expression levels of VEGF-A165 in conditioned medium in HepG2 cells transfected with silencing RNA of SP1 (SiSP1), EGR1 (SiEGR1), Nrf2 (SiNrf2), SIRT1 (SiSIRT1), PGC-1 (SiPGC-1) and control (SiC) at each glucose concentration (4.5 g/L or 0.1 g/L) under hypoxic conditions (<1.0% O2) for 24 h. (n=3, mean ± SD; ns, not significant; *P < 0.05, **P < 0.01). Additional file 6. (a): VEGF-A165 mRNA expression under hypoxic cond [file 12885_2026_16069_MOESM1_ESM.zip › SiHIF Gels and Blots Image..pptx]

## Slide 1
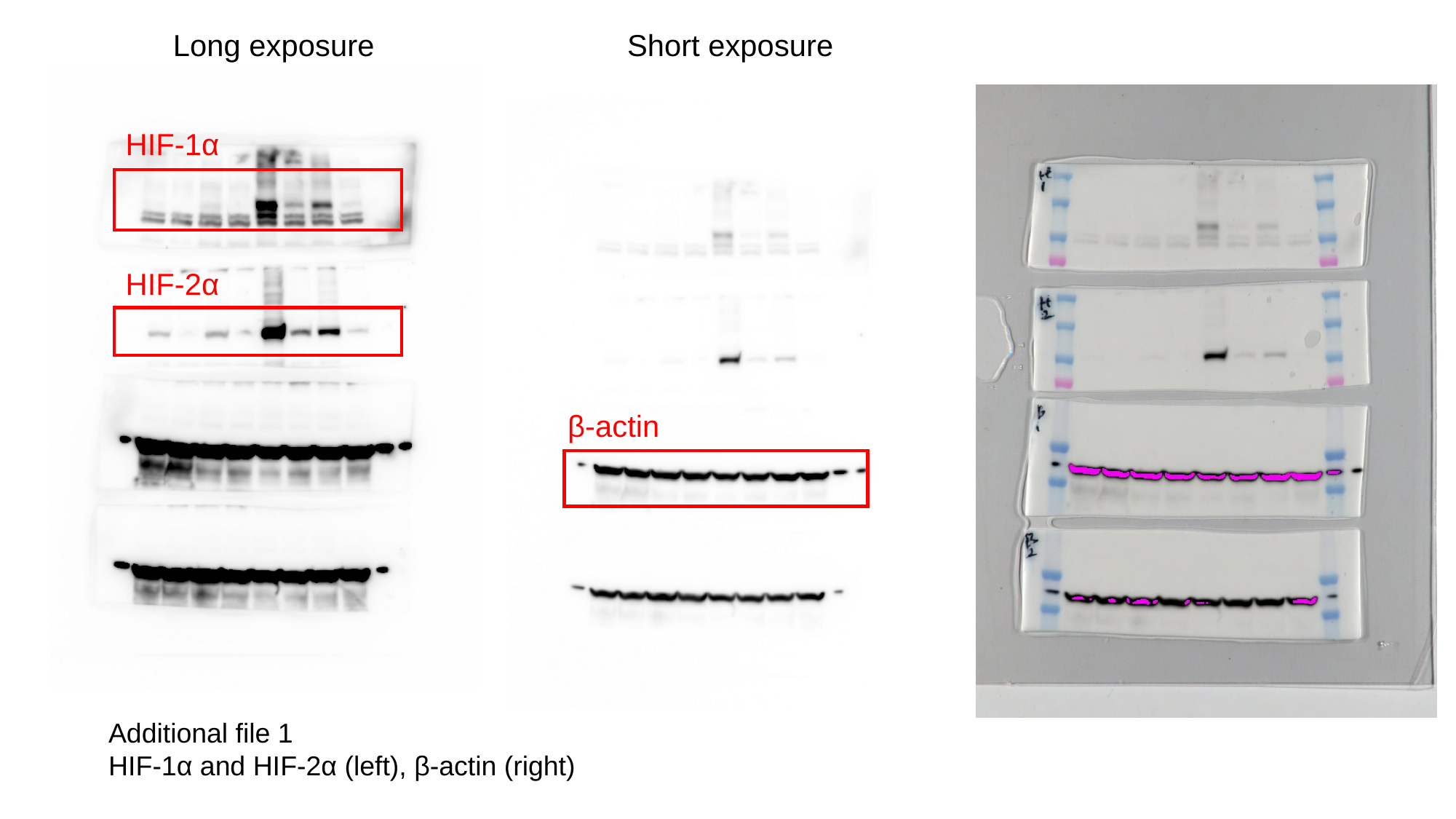

Long exposure
Short exposure
HIF-1α
HIF-2α
β-actin
Additional file 1
HIF-1α and HIF-2α (left), β-actin (right)
